# NOX4 downregulation leads to senescence of human vascular smooth muscle cells

**DOI:** 10.18632/oncotarget.12079

**Published:** 2016-09-16

**Authors:** Dorota Przybylska, Dorota Janiszewska, Aleksandra Goździk, Anna Bielak-Zmijewska, Piotr Sunderland, Ewa Sikora, Grażyna Mosieniak

**Affiliations:** ^1^ Laboratory of Molecular Bases of Aging, Department of Biochemistry, Nencki Institute of Experimental Biology of Polish Academy of Sciences, Warsaw, Poland

**Keywords:** senescence, NOX4, VSMC, ROS, Gerotarget

## Abstract

Senescence is a stress response characterized by an irreversible growth arrest and alterations in certain cell functions. It is believed that both double-strand DNA breaks (DSB) and increased ROS level are the main culprit of senescence. Excessive ROS production is also particularly important in the development of a number of cardiovascular disorders. In this context the involvement of professional ROS-producing enzymes, NADPH oxidases (NOX), was postulated. In contrary to the common knowledge, we have shown that not only increased ROS production but also diminished ROS level could be involved in the induction of senescence.

Accordingly, our studies revealed that stress-induced premature senescence (SIPS) of vascular smooth muscle cells (VSMCs) induced by doxorubicin or H_2_O_2_, correlates with increased level of DSB and ROS. On the other hand, both SIPS and replicative senescence were accompanied by diminished expression of NOX4. Moreover, inhibition of NOX activity or decrease of NOX4 expression led to permanent growth arrest of VSMCs and secretion of interleukins and VEGF. Interestingly, cells undergoing senescence due to NOX4 depletion neither acquired DSB nor activated DNA damage response. Instead, transient induction of the p27, upregulation of HIF-1alpha, decreased expression of cyclin D1 and hypophosphorylated Rb was observed. Our results showed that lowering the level of ROS-producing enzyme - NOX4 oxidase below physiological level leads to cellular senescence of VSMCs which is correlated with secretion of pro-inflammatory cytokines. Thus the use of specific NOX4 inhibitors for pharmacotherapy of vascular diseases should be carefully considered.

## INTRODUCTION

Cellular senescence is a stress response that leads to irreversible cell cycle arrest accompanied by a set of distinct phenotypic and functional changes. On the basis of these changes senescent cells could be recognized both *in vitro* and *in vivo* [1, 2]. Senescent cells accumulate with age in a variety of tissues in a number of different organisms including mouse, primates and humans [3, 4]. The involvement of cellular senescence in both physiological and pathological processes has been documented. The general biological role of senescence is to eliminate damaged or unwanted cells, however, the outcome of it could be either beneficial or detrimental depending on the cellular and tissue context [5]. There are a number of triggers that lead to cellular senescence. One of the most commonly recognized is telomere shortening that progresses gradually with each cell division and leads to so-called replicative senescence [6]. In contrast to gradual exhaustion of proliferation potential, cellular senescence could also be induced within short time by stress factors [7]. Among these factors ROS have been considered as the most common. During the past decades the harmful effect of excessive ROS production has been linked to damage of macromolecules among which DNA damage is considered as the most relevant to the induction of senescence. Accordingly, the increased ROS level was observed due to action of different prosenescent stimuli such as DNA damaging agents, oncogenes and loss of telomere-protective functions [8]. Increased level of ROS accompanies organismal aging as well as age-related diseases further indicating a causal link. Since 1956 when Harmans’ free radical theory of aging was formulated [9], ROS were considered as a toxic by-products of dysfunctional mitochondria that drives the aging process on the cellular, tissue and organ level. However, recent studies have revealed a beneficial effect of ROS action. Namely, ROS could be actively generated in cells and mediate intracellular signalling acting as secondary messengers. ROS have been shown to activate or inhibit kinases, phosphatases as well as transcription factors involved in regulation of prosurvival pathways, proliferation, differentiation and metabolism [10, 11].

Along with the controversies of the casual link between ROS production and aging, there is still an open question concerning the role of ROS in cell senescence. It was suggested that ROS produced by mitochondria in a retrograde way induce nuclear DNA damage from which the signal is further transduced to finally elicit cell senescence [12]. Recently a few publications have linked NADPH-dependent oxidase, NOX4 with the process of cellular senescence. It was shown that increasing expression of NOX4 and production of ROS in endothelial cells induce oxidative DNA damage as well as mitochondria dysfunction that promote replicative senescence of these cells [13, 14]. The involvement of NOX4 in oncogene-induced senescence has also been described [15, 16, 17]. Increased expression of Nox4 was found in smooth muscle cells present in the aortas of aged rats [18] as well as in mouse senescent smooth muscle cells from atherosclerotic plaques [19].

NOX4 is a member of NADPH oxidase family, which comprises seven proteins, namely NOX1-5 and DUOX1,2. They are characterized by distinct tissue and cell compartment distribution and mechanism of activation [20]. NOX4 is one of the isoforms that is expressed in different cell types such as osteoblasts, preadipocytes, keratinocytes and neurons. It is also found in vasculature, namely in endothelial and vascular smooth muscle cells [21]. This oxidase is unique in that it appears to be constitutively active and produces predominantly H_2_O_2_, as a consequence of a specific alteration in its E-loop [22]. In vascular smooth muscle cells, NOX4 was initially described as a key regulator of cellular differentiation and quiescence [23, 24] which suggested its homeostatic function. Subsequently it was shown that NOX4 contribute to vascular smooth muscle cell proliferation, migration and, under certain conditions, hypertrophy [25, 26] that are important in arterial remodelling and atherogenesis. However, excessive activation of NOXs, resulting in an increased production of ROS, was shown to promote the development and progression of cardiovascular diseases [27, 28]. Thus, NOX4 was shown to exert both a beneficial as well as detrimental effect, depending on the cell context and stimuli that influence its activity.

The aim of these studies was to investigate the role of NOX4 in the senescence of human vascular smooth muscle cells. The obtained results reveal that both replicative as well as stress-induced senescence of human VSMCs correlates with downregulation of NOX4 expression. Moreover, treatment of cells with DPI, a known inhibitor of flavoenzymes, as well as silencing of NOX4 lead to decreased production of ROS and induction of permanent growth arrest characterized by typical markers of senescence. Our results provide evidence of a crucial role of NOX4 in the regulation of proliferation of human VSMCs and prove that, similarly to excessive ROS production, reduced level of ROS can lead to induction of an irreversible state of growth arrest - the senescence.

## RESULTS

### hVSMCs undergo stress-induced premature senescence (SIPS) upon doxorubicin and hydrogen peroxide treatment

**Figure 1:**
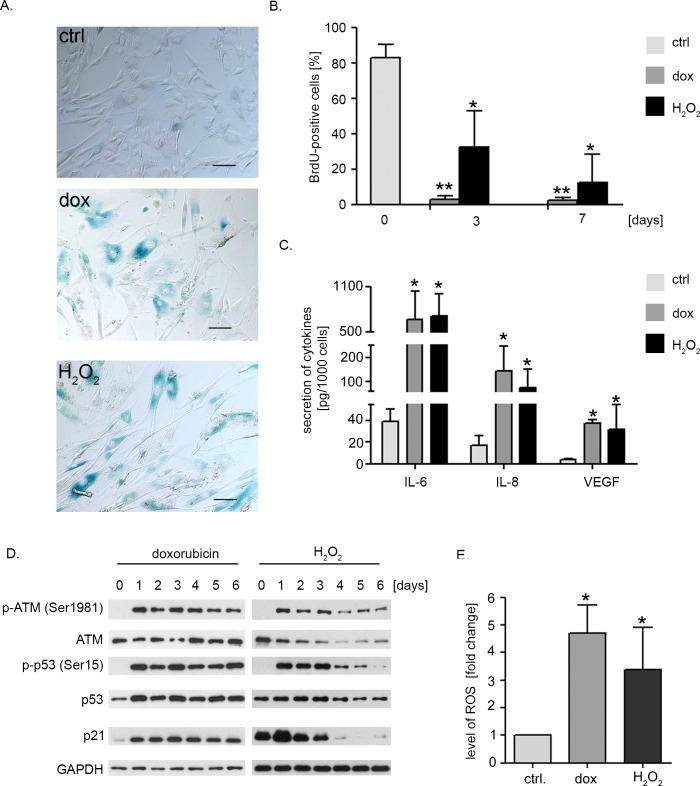
Doxorubicin or hydrogen peroxide treatment induce senescence of hVSMCs **A.** Detection of SA-β-gal activity in non-treated (ctrl), dox- and H_2_O_2_-treated cells. Low passage cells (passage number between 4 and 10) were treated either with dox (1 μM) for 2 hour or with a single dose of H_2_O_2_ (100 μM). The SA-β-gal activity was analysed after 6 days of a culture. The bars indicate 100 μm. **B.** Analysis of proliferation potential estimated by BrdU incorporation test. Cells were either untreated (control) or treated with dox or H_2_O_2_ and incorporation of BrdU was analysed after 6 days; graph presents mean ±SD from at least three independent experiments. **C.** Analysis of the level of SASP factors (IL-8, IL-6, VEGF) secreted by control (young) *versus* senescent cells, mean (±SD) level of secretion of selected cytokines was calculated from three independent experiments. **D.** Western blot analysis of the level of selected DDR proteins - ATM, p53 and p21 in hVSMCs undergoing senescence upon dox or H_2_O_2_ treatment. Representative blots from three independent experiments are shown. **E.** The relative level of ROS in hVSMCs that underwent senescence upon doxorubicin or H_2_O_2_ treatment. Low passage cells (passage number 4-10) were treated with dox or H_2_O_2_ as described and the level of ROS was estimated by flow cytometry measurement of carboxy-H_2_DCFDA fluorescence. Graphs present the mean fluorescence intensity (±SD) from 4 independent experiments.

In order to study the influence of dox or H_2_O_2_ on intensively proliferating low passage hVSMCs, cells were treated with 1 μM doxorubicin for 2 hours or with one dose of 100 μM H_2_O_2_ and cultured for several days. Microscopic observation revealed that within few days cells changed their morphology and became bigger and more flattened than control ones. Qualitative analysis performed 6 days after treatment proved that majority of dox- and H_2_O_2_-treated cells had increased activity of SA-β-gal (Figure 1A). We observed also a significant decrease in proliferation as a gradual decrease in the number of BrdU-incorporating cells (Figure 1B). Importantly, we did not observe a cell death after treatment of the cells with dox or H_2_O_2_ (suppl. Figure 1).

As one of the features of senescence is the senescence-associated secretory phenotype (SASP) we investigated the level of interleukin 6 (IL-6), 8 (IL-8) and vascular endothelial growth factor (VEGF) secreted after 6 days of hVSMC treatment with dox or H_2_O_2_ (Figure 1C). Increased level of all three cytokines was detected in the medium collected from cultures of senescent cells compared to control ones. The level of IL-6 was the highest as we observed 17-fold increase of this cytokine in the medium collected from dox- and H_2_O_2_-treated cell culture. Secretion of IL-8 was increased 9 (dox-treated cells) and 5 times (H_2_O_2_-treated cells). Although prematurely senescent hVSMCs secreted 10 times more VEGF than control cells, the concentration of this cytokine in the medium was the lowest among the investigated factors.

DNA damage response (DDR) pathway is considered to be the main pathway responsible for the induction of senescence. Thus the level of expression and activation of selected DDR proteins was checked (Figure 1D). After one day of treatment we observed autophosphorylation of ATM kinase on Ser1981 and phosphorylation of p53, a downstream target of ATM, on Ser15. Activation of these proteins in dox-treated cells lasted for at least 6 days whereas in H_2_O_2_-treated cells this phenomenon was transient as we observed a gradual decrease of phospho-p53 as well as total p53 after the 3^rd^ day of the experiment. The level of p21, a cyclin-dependent kinase inhibitor which is the main downstream target of p53 was also increased.

As senescence of cells is often related to increased level of ROS, we measured the intracellular ROS level using carboxy-H_2_DCFDA. Seven days after treatment with dox or H_2_O_2_ we observed 4.6- and 3.3-fold increase in the level of ROS in comparison to control cells (Figure 1E).

### hVSMCs undergoing senescence have decreased level of *NOX4*

**Figure 2:**
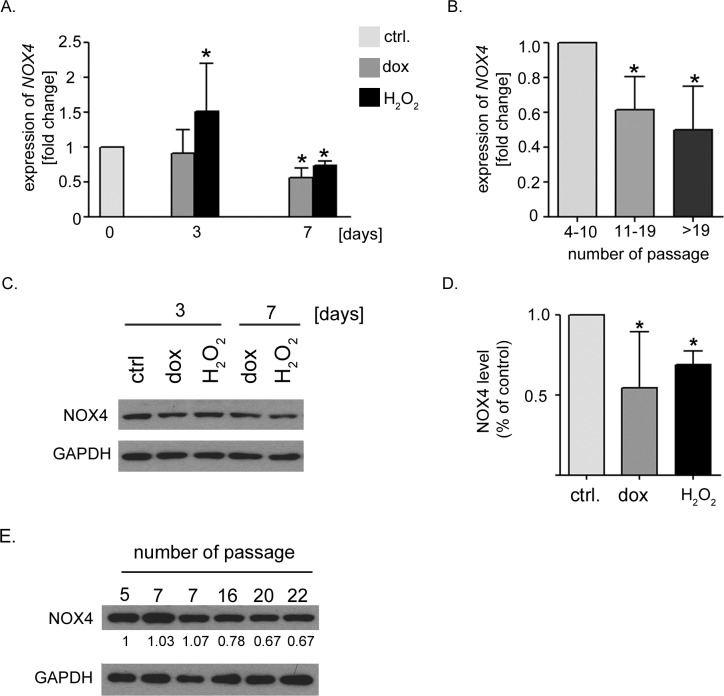
Decreased expression of NOX4 in hVSMCs undergoing senescence **A.** The relative level of *NOX4* in the cells undergoing senescence upon doxorubicin or H_2_O_2_ treatment. The level of expression was normalized to *ACTB* expression and related to the control cells. Graph presents the mean ±SD from at least three independent experiments. **B.** The relative level of *NOX4* during replicative senescence. The level of *NOX4* expression was normalized to *ACTB* expression and related to the control cells. Graph presents the mean value of *NOX4* level (±SD) from at least three independent experiments. **C.** The representative blot presenting NOX4 protein level detected in the cells induced to senescence by dox or H_2_O_2_ treatment. **D.** A densitometric analysis of the level of NOX4 protein in the cells treated with dox or H_2_O_2_ for 7 days. Graph presents a mean (±SD) value from 3 independent experiments. **E.** The representative blot presenting NOX4 protein level detected in the cells undergoing replicative senescence. The numbers show the relative level of NOX4 protein calculated on the base of densitometric analysis.

Increased level and activity of NOX4 was shown to be relevant for the induction of replicative senescence of endothelial cells as well as oncogene-induced senescence of fibroblasts [13, 15]. As NOX4 is constitutively active [29] and regulated mainly on the transcriptional level we estimated the level of *NOX4* mRNA in hVSMCs undergoing senescence. We took advantage of the model of replicative senescence of hVSMCs previously characterized by us [30] and compared *NOX4* level in the cells at early and late passages as well as in the cells undergoing SIPS. Interestingly, we observed that downregulation of *NOX4* expression as well as decrease of a protein level accompanied both stress-induced premature senescence and replicative senescence (Figure 2). Moreover, we performed analysis of *Nox4* expression in rat VSMCs undergoing senescence upon dox and H_2_O_2_ treatment. Although rat VSMCs did not undergo replicative senescence (not shown) we were able to reveal that they remained sensitive to the induction of senescence by both tested agents (suppl. Figure 2A, 2B, and 2C). We noticed upregulation of the expression of *Nox4* in rat cells undergoing senescence (suppl. Figure 2D) which suggests that the role of NOX4 oxidase in the induction of senescence could differ between human and rodent VSMCs.

### Inhibition of NOXs by DPI induces senescence of hVSMCs

To verify whether inhibition of NOX4 activity induces senescence we treated hVSMCs with DPI, a non-specific flavoenzymes inhibitor which is also used for revealing the role of NADPH oxidase family members. We observed that one day treatment with DPI used in a wide range of concentrations (from 10 nM to 1.25 μM) led to an inhibition of proliferation (data not shown). Moreover, significant reduction of the level of ROS was observed after 3 days of cell treatment with DPI (suppl. Figure 3A). Cell cycle analysis revealed that at the same time there was statistically significant reduction of the percentage of the cells in the S phase while the number of cells in the G2/M phase increased almost two times comparing to control cells (suppl. Figure 3B).

To further investigate whether DPI exerts a prolonged effect on the growth of hVSMCs the BrdU incorporation test was performed. Three days after DPI treatment only 4% of cells was able to synthesize DNA. Interestingly, during the next four days of culture in DPI-free medium cells were unable to resume proliferation since no increase in BrdU-positive cells was observed (suppl. Figure 3C). Inhibition of proliferation of hVSMCs upon DPI treatment was accompanied by increased activity of SA-β-gal (suppl. Figure 3D). We observed that 7 days after treatment with DPI majority of cells were SA-β-gal-positive in comparison to non-treated cells. Importantly, DPI treatment did not lead to cell death since we did not observe decrease of the number of the cells till the last day of the experiment (suppl. Figure 3E).

### Downregulation of *NOX4* leads to senescence of hVSMCs

**Figure 3:**
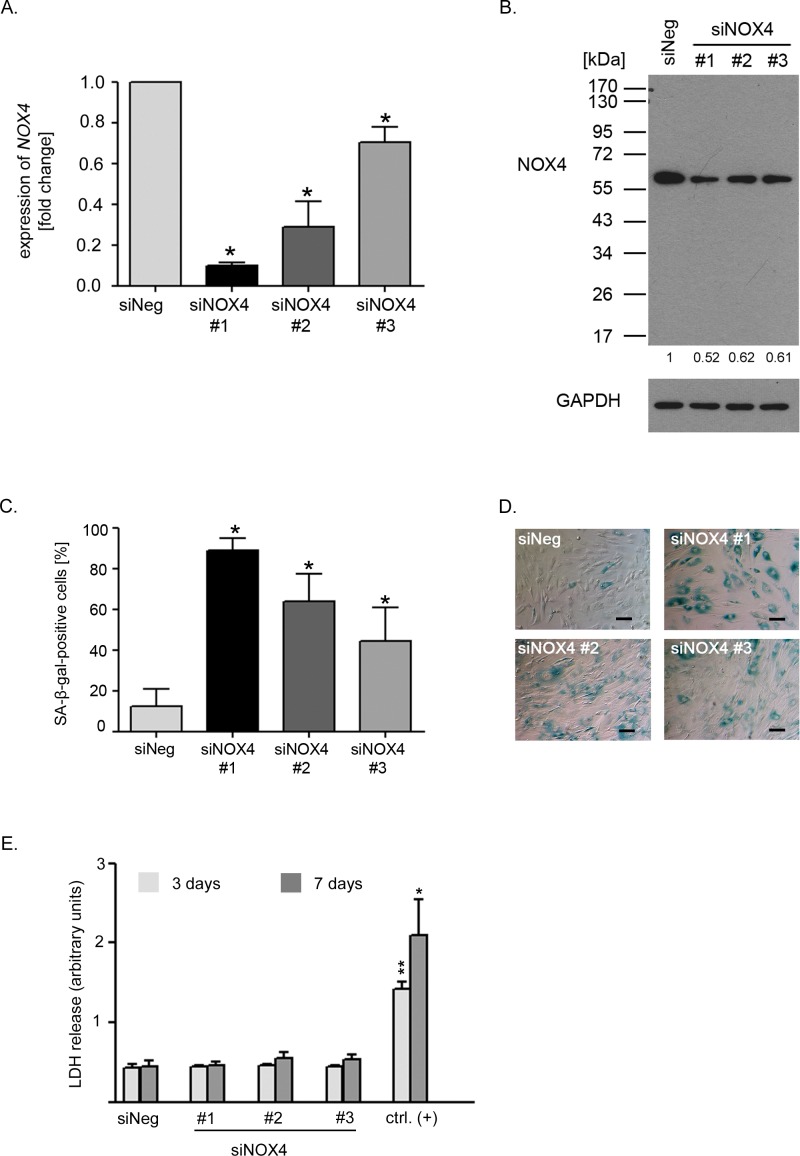
Reduced NOX4 level lead to cellular senescence of VSMCs **A.** The relative level of *NOX4* in the cells transfected with 3 different specific siRNAs against *NOX4* (siNOX4). The level of expression was normalized to *ACTB* expression and related to the non-targeting siRNA-transfected cells (siNeg). Graph presents the mean ±SD from at least three independent experiments. **B.** NOX4 protein level estimated 48 hours after transfection with three different siRNAs against *NOX4*. Western blot analysis confirming the specificity of antibody used for detection of this protein. Detection was performed on the whole blot and the positions of MW markers were indicated. **C.**, **D.** Estimation of SA-β-gal activity in the non-targeting siRNA-transfected cells (siNeg) and the cells transfected with 3 different specific siRNAs against *NOX4*. The graph presents the mean percentage (±SD) of SA-β-gal-positive cells calculated from at least three independent experiments. The bars on the photographs indicate 100 μm. **E.** Analysis of LDH release by the cells transfected with 3 different *NOX4*-targeting siRNAs or with non-targeting sequence (siNeg). For positive control low passage VSMCs were treated with lysis solution according to manufacturer's recommendation. The level of LDH release was measured 3 and 7 days after transfection. Graph presents data from 3 independent experiments, mean±SD. For statistical analysis the level of LDH release from the cells transfected with NOX4 siRNA or treated with lysis solution were compared with Neg siRNA -transfected cells at each time point.

As DPI is a non-specific inhibitor of NADPH oxidases, we reduced *NOX4* level using specific siRNA. The efficacy of *NOX4* downregulation differ between sequences used for a transfection. The lowest level of *NOX4* transcript, below 10% of that in non-targeting siRNA-transfected cells (siNeg), was obtained with sequence 1 (#1) while the remaining two sequences (siNOX4#2 and siNOX4#3) reduced NOX4 transcript to about 30% and 70% of the control level, respectively (Figure 3A). Accordingly we observed decreased level of NOX4 protein upon NOX4 gene silencing, which was reduced to approximately 52% of the NOX4 level detected in the cells transfected with non-targeting siRNA (Figure 3B).

**Figure 4:**
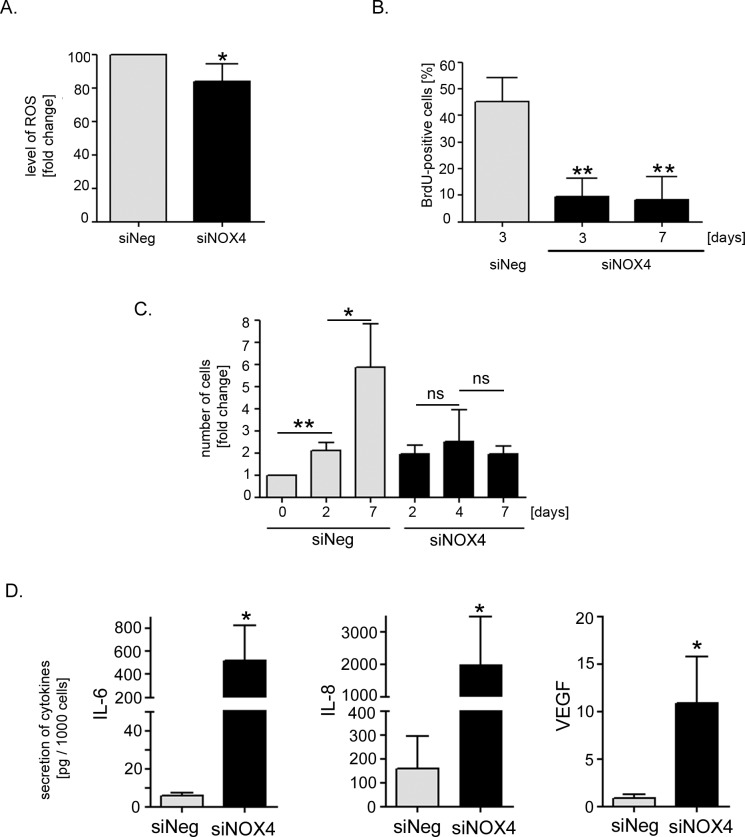
Estimation of the level of ROS, markers of proliferation and secretory phenotype of the cells with silenced *NOX4* expression **A.** The relative level of ROS in hVSMCs analysed 48 hours after transfection with siNOX4#1 (siNOX4). The level of ROS was estimated by flow cytometry measurement of carboxy-H_2_DCFDA fluorescence. Graph presents the mean fluorescence intensity (±SD) from three independent experiments of siNOX4-transfected cells related to the level of fluorescence of siNeg-transfected cells. **B.** Analysis of proliferation potential of siNOX4 transfected cells based on DNA synthesis capacity. BrdU incorporation test was performed on the 3^rd^ and 7^th^ day after transfection. The graph presents the mean percentage (±SD) of BrdU-positive cells from 3 independent experiments. **C.** Analysis of growth rate of siNOX4- and siNeg- transfected cells based on cell counting. Graph presents a fold change (mean value ±SD) calculated from at least three independent experiments. **D.** Analysis of the level of SASP factors (IL-8 and IL-6) secreted by siNeg- and siNOX4-transfected cells. The mean level (±SD) of selected cytokine secretion was measured in three independent experiments 7 days after transfection.

Interestingly, low level of *NOX4* expression correlated with the inhibition of cell proliferation and with the induction of senescence. The number of SA-β-gal-positive cells correlated inversely with the level of NOX4 expression obtained after transfection with different siRNA sequences (Figure 3C, 3D). Importantly, NOX4 silencing did not lead to the cell death (Figure 3E). For further experiments the siNOX4#1 sequence (siNOX4) was chosen. Accordingly we measured changes in the intracellular level of ROS upon NOX4 silencing. We observed statistically significant reduction of ROS by nearly 20% of the level measured in the control cells (Figure 4A). Moreover, cells with diminished *NOX4* expression cease to proliferate since a significant decrease in the number of cells able to replicate DNA was observed already 3 days after transfection. These cells did not restart DNA replication during the next 4 days of the culture (Figure 4B). This observation was further confirmed by cell counting which showed that there was no significant increase in the number of the cells upon NOX4 silencing (Figure 4C). Inhibition of proliferation and increased activity of SA-β-gal were accompanied by a secretion of proinflammatory proteins, confirming the presence of SASP. Interestingly, 7 days after transfection we observed a 90-fold increase in the secretion of IL-6 and a 12-fold increase in the secretion of IL-8 in comparison to the cells transfected with siNeg (Figure 4D).

### NOX4-depleted cells undergo senescence without DDR activation

**Figure 5:**
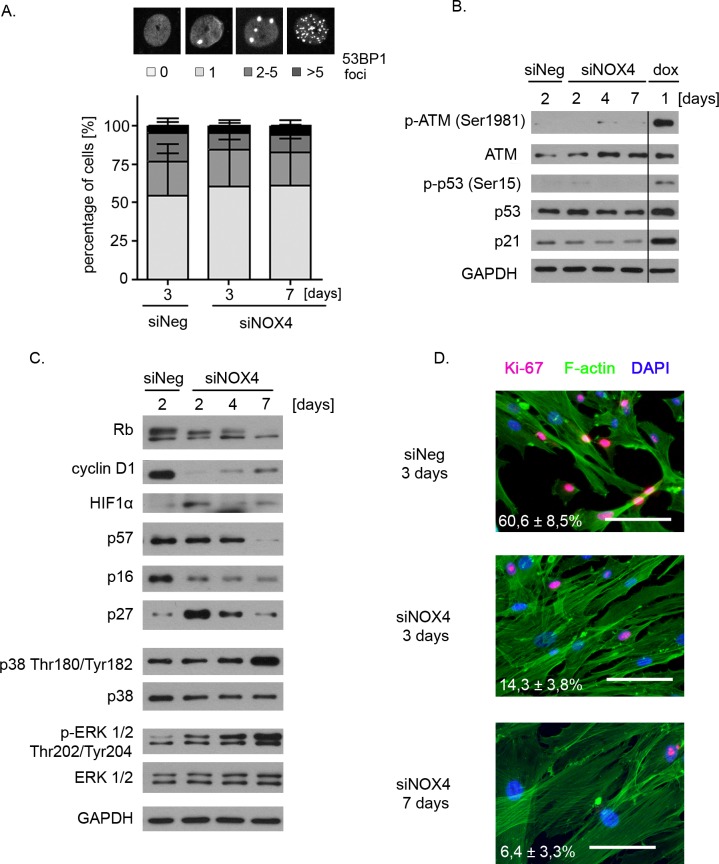
Molecular determinants of NOX4-downregulation-induced senescence **A.** Analysis of the number of 53BP1 foci in the cells transfected with siNeg and siNOX4 (3 and 7 days after transfection). Cells were classified into one of four groups depending on the number of foci present in their nuclei (group 1: lack of foci in the nucleus; group 2: 1 focus; group 3: 2 to 5 foci; group 4: more than 5 foci per nucleus). Graph presents the mean number ±SD of cells with particular number of 53BP1 foci calculated from three independent experiments. **B.** Western blot analysis of the level of selected DDR proteins - ATM, p53 and p21 in hVSMCs undergoing NOX4-downregulation-induced senescence. 1-day dox-treated cells served as a positive control of the activation of the DDR pathway. Representative blots from three independent experiments are shown. **C.** Western blot analysis of the level of the cell cycle proteins, hypoxia inducible factor 1α and extracellular signal kinase (ERK) in the cells transfected with siNeg and siNOX4 (days 0 to 7). Representative blots from three independent experiments have been shown. **D.** Immunocytochemical detection of the proliferation marker Ki-67 in the cells transfected with siNeg or siNOX4. The bars indicate 100 μm.

The most common trigger of the senescence is DNA damage leading to activation of the DDR pathway which was shown to be responsible for both inhibition of proliferation and acquisition of the secretory phenotype. Thus we analysed 53BP1 foci formation which mark DNA breaks as well as the level of proteins of the DDR pathway in VSMCs transfected with NOX4 siRNA. As expected, there was no difference in the number of 53BP1 foci between control cells transfected with siNeg and the cells with reduced NOX4 level (Figure 5A). Western blotting analysis revealed that there was no activation of ATM, p53 and no accumulation of p21. On the contrary, phosphorylation of ATM Ser1981, p53 Ser15 and upregulation of p21 was easily detected in dox-treated cells which served as a positive control (Figure 5B). Thus, the obtained results prove that DDR is not responsible for induction of senescence of hVSMCs upon NOX4 silencing.

Instead, the Western blotting analysis (Figure 5C) revealed a decrease in cyclin D1 level and the accumulation of a hypophosphorylated form of Rb. NOX4 silencing was accompanied by the induction of HIF1α protein. Moreover, transient upregulation of p27, a cell cycle inhibitor, was observed upon NOX4 silencing while the level of p57, p16 (Figure 5C) and p14­­ (data not shown) remained unchanged or declined after transfection. Finally, we detected a gradual decrease in the number of Ki-67-positive cells from about 60% in the control cell population down to less than 7% in siNOX4-transfected hVSMCs at day 7 (Figure 5D), proving that NOX4-silencing led to a complete loss of the proliferation potential of VSMCs.

Moreover, we observed strong activation (phosphorylation) of ERK1/2 in the cells with diminished NOX4 level. The increased ERK1/2 phosphorylation was observed already after 2 days of NOX4 silencing and remained at a very high level when cells expressed the markers of senescence. 7 days after transfection we also observed an increased level of p38 phosphorylated on Thr180/Tyr182 which correlated with the acquisition of SASP.

## DISCUSSION

The data presented here show for the first time that the decreasing of the expression of ROS-producing NADPH oxidase, NOX4 in human vascular smooth muscle cells leads to permanent growth arrest. Obtained results indicate that lowering ROS production below the physiological level could drive the senescence of normal cells. The importance of oxidative stress in the development of vascular pathologies is well recognized. For instance, the role of mitochondrial-derived ROS in regulation of the NF-κB pathway and corresponding age-related inflammatory activation of endothelium was reported [30]. Besides that, senescence of cells building the vasculature in the development of atherosclerosis was described. Senescent VSMCs participate in the formation of unstable plaques. The low number of VSMCs as well as diminished amount of collagen present in the cap of these plaques makes them vulnerable to rapture. Moreover, growth arrested VSMCs significantly restrict the repair capacity of the plaque, which contributes to high risk of myocardial infarction and stroke [32, 33, 34].

The link between ROS production and senescence seems to be unquestionable. The causative role of ROS in the induction of both stress-induced and replicative senescence of human fibroblasts was previously demonstrated [35, 36]. Conversely, attenuation of oxidative stress, either by culturing cells under hypoxic conditions or treatment with antioxidants, delayed senescence and increased proliferation potential [37, 38]. Increased ROS production drives senescence by activating redox-sensitive signalling pathways. Alternatively, the prosenescence activity of ROS may arise from the deleterious effect of damage inflicted to cellular macromolecules. In this context, a spectrum of DNA damages seems to be particularly relevant. Among them double-strand DNA breaks (DSB) are considered to play a major role in senescence induction. Upon DSB cells activate the DNA damage response (DDR) i.e., a cascade of proteins, which enable recognition of DNA damage (e.g. γH2AX, 53BP1) or transduce the signal (like ATM/ATR and CHK kinases) to downstream effectors - p53/p21 [39, 40]. Accordingly, we have shown that VSMCs exposed to either H_2_O_2_ or a DNA damaging drug - doxorubicin, acquire DSB, activate DDR and undergo stress-induced premature senescence (SIPS). Moreover, we found that increased ROS production accompanies both SIPS and replicative senescence suggesting ROS involvement in senescence of VSMCs. Although the main source of ROS in cell is the mitochondrial respiratory chain the involvement of which in aging is well documented [41], an important role of professional ROS producing enzymes such as NADPH oxidases has been postulated [42] and gained much attention. Increased NOX4 level has been found in the vascular smooth muscle cells from aortas of old rats [18]. Recently, a higher level of NOX4 expression in human VSMCs obtained from older individuals has also been reported [43]. Moreover, Lener et al. [13] have shown that increased NOX4 activity causes oxidative damage in endothelial cells isolated from the human umbilical vein (HUVECs), and leads to the loss of replicative potential. Expression of NOX4 was also elevated during oncogene-induced senescence. Accordingly it was described that ROS produced by this oxidase acts as mitogenic signalling molecules that fuel oncogene-driven aberrant cell proliferation that leads to the replication stress and consequently DDR activation [44]. Moreover, NOX4 participates in the induction of so-called ‘bystander senescence’ as shown by Hubackova et al. [45]. Namely, increased level of Nox4 mRNA was detected in fibroblasts treated with conditioned medium collected from the cells that underwent replicative, oncogene- or drug-induced senescence. Induction of Nox4 expression was dependent on high level of IL-1 and TGF-β secreted by senescent cells. Thus it was surprising for us to find out that increased level of ROS observed in VSMCs undergoing replicative or stress-induced premature senescence is not correlated with increased level of NOX4 oxidase. In contrary, we observed slight decrease of its expression, questioning the role of NOX4 oxidase in triggering the oxidative stress and consequently DNA damage, which was detected in senescent VSMCs. Furthermore, induction of senescence upon NOX4 depletion in human VSMCs described herein suggests that decreased expression of NOX4 could force cells to permanent growth arrest. However more studies is needed to reveal the role of NOX4 in replicative and stress induced senescence of VSMCs.

Taking into account the important role of oxidative stress in the development of atherosclerosis, the involvement of particular NADPH oxidases, as professional ROS producing enzymes was intensively studied. However, the data are contradictory and suggest both atheroprotective and pro-atherosclerotic role of NOX4 in humans and mice [43, 46, 47]. Of note, cell senescence was not considered in these studies.

The causal link between NOX4, atherosclerosis progression and senescence was postulated by Xu et al. [19]. Upregulation of Nox4 expression was revealed in SMCs isolated from the atherosclerotic plaques of ApoE^−/−^ LDLR^−/−^ mice. Moreover those cells were growth arrested and forced expression of Nox4 in proliferating mouse SMCs caused senescence. Accordingly, we have also found upregulation of Nox4 expression in VSMCs, isolated from rat aortas, that undergo SIPS. Since this result is contradictory to what was observed in human VSMCs subjected to the same stress conditions, we feel entitled to claim that Nox4 expression might be differently regulated in rodent and human VSMCs undergoing senescence.

The main culprit of cellular senescence is induction of DNA damage. However, as expected, we showed that neither DNA damage nor DDR participate in senescence induced by downregulation of NOX4 expression. Instead, we observed transient upregulation of p27, which correlates with inhibition of NOX4 expression and lower level of ROS. No induction of other cell cycle inhibitors, namely p21, p57 and p16 was observed. Of note, it was previously shown that culturing the cells under hypoxic conditions led to growth arrest mediated by cell cycle inhibitors - p27 [48], p27 and p21 [49] or p21 alone [50]. The p21 and p27 was shown to be increased by the HIF1α transcription factor [49] or by a mechanism that is independent of HIF1α transcriptional activity but still dependent on its expression [50]. Apart from the induction of cell cycle inhibitors under hypoxic conditions, HIF-1α has been shown to directly bind to the gene promoter region of cyclin D1 and inhibits its expression [51, 52]. Interestingly, we demonstrated slight upregulation of HIF-1α upon NOX4 silencing. HIF1α, expression correlated with downregulation of cyclin D1 level and gradual accumulation of the hypophosphorylated form of Rb. The activated signalling pathway ends up in an irreversible loss of the proliferation potential as shown by a decrease in the number of cells expressing Ki-67 and secretion of selected SASP factors by VSMCs induced to senescence by inhibition of NOX4 expression. Of note, several studies have shown that the level of cyclin D1 is increased in senescent human fibroblasts [53] and vascular smooth muscle cells [54]. However cyclin D1 is also necessary for the progression from G1 to S phase. It was demonstrated that overexpression of cyclin D1 shortens G1 phase and enhance proliferative capacity leading to tumorigenicity of rodent fibroblasts [55]. In contrary, silencing of this cyclin resulted in inhibition of proliferation of cancer cells [56, 57]. We suspect that in NOX4-depleted cells downregulation of cyclin D1 results in permanent growth arrest. Lack of the important G1 cyclin is sufficient to activate pRb (hypophosphorylation) without involvement of p16 inhibitor which acts on cyclin D1-CDK4/6 complex.

We can speculate that diminished expression of NOX4 in VSMCs which leads to decreased production of NOX4-derived ROS mimics hypoxic conditions and induces senescence. Interestingly, it has been shown recently that NOX4 is an oxygen-sensing enzyme which activity depends on pO_2_. This allows NOX4 to generate H_2_O_2_ as a function of oxygen concentration throughout a physiological range of pO_2_ values and to respond rapidly to changes in pO_2_ [58]. NOX4 regulation by oxygen seems to be more complex. NOX4 expression was shown to increase in human lung microvascular endothelial cells exposed to hyperoxia [59] but upregulation of NOX4 expression was also observed in human pulmonary artery smooth muscle cells cultured under low concentration of O_2_. Thus, regulation of NOX4-madiated signalling by oxygen may be complex and tissue-dependent.

The causative role of hypoxia in senescence induction has recently been shown. Mo et al. [60] identified senescent melanoma cells located mainly around necrotic areas of tumours from C57BL/6J, where hypoxic conditions prevails. Moreover, culturing of melanoma cells in the presence of CoCl_2_ which mimics hypoxic conditions, led to senescence of those cells. In contrast, hypoxia was also reported to promote proliferation and inhibit senescence [61] or to suppress the conversion from proliferative arrest to cellular senescence [62, 63]. This contradictory observations may result from different degree of hypoxia in different cellular contexts and unveil the complexity of regulation of ROS-mediated signalling pathways.

Interestingly, Shimi et al. (2011) [64] have also demonstrated that decreased ROS level could participates in the induction of senescence observed after downregulation of the expression of lamin B1 in fibroblasts. However it was shown that the decrease in the ROS level depends on p53 upregulation. The authors suggested that increased level of p53 could lead to the induction of antioxidant gene expression that influenced ROS level. In contrary, we did not observe p53 upregulation in NOX4 silencing-induced senescence. Thus, we can assume that decreased level of ROS in the cells with downregulated NOX4 is a direct consequence of the lower level of a ROS-producing enzyme. Still further studies are needed to reveal the exact molecular mechanism that drives cellular senescence upon NOX4 depletion in proliferating VSMCs.

Summarizing, we have demonstrated the critical role of NOX4 in regulation of proliferation of human VSMCs. Downregulation of NOX4 expression contributes to senescence of smooth muscle cells, which potentially could increase local inflammation. Since specific NOX isoform inhibitors are considered as potential drugs for treatment of vascular pathologies, the possibility that they may induce senescence of cells building the vasculature should be taken into account.

## MATERIALS AND METHODS

### Ethics statement

Investigation has been conducted in accordance with the ethical standards and according to the Declaration of Helsinki and according to national and international guidelines and has been approved by the Ist Ethical Committee in Warsaw, Poland.

### Cells and treatment

Human primary vascular smooth muscle cells (hVSMCs) were purchased from Lonza (Switzerland) and ATCC (LGC Standards) and cultured in SMGM (Smooth muscle growth medium; Lonza). Rat smooth muscle cells (rVSMCs) were isolated from aortas of 3 month old rats (Wistar) and cultured in DMEM (Sigma Aldrich Poznan, Poland). Both types of cells were cultured in 37°C in humidified atmosphere containing 5% CO_2_.

For experiments, proliferating cells (cells between passage 4 to 10) were seeded at a density of 3000 cell/cm^2^ 24 h before treatment or transfection. Cells were treated with 1μM doxorubicin (dox) for 2 h and then cultured in a compound-free medium, treated with 100 μM hydrogen peroxide (H_2_O_2_) or with 1.25 μM diphenyleneiodonium chloride (DPI; only hVSMCs). Every third day the medium was changed for a fresh one.

To obtain replicatively senescent cells (only hVSMCs), cells were passaged every four days or when confluent, until they cease to proliferate. Each time cells were counted to determine the number of population doublings and cumulative population doublings (cPD) value. Cells were considered as replicatively senescent when cPD reached a plateau.

### LDH release analysis

LDH release was measured in a medium collected from the cell culture 1, 3 and 7 days after cells treatment. The measurements were performed using CytoTox96^®^ Non-Radioactive Cytotoxicity Assay (Promega), according to a manufacturer's protocol. For positive control low passage number VSMCs were treated with lysis solution according to manufacturer's recommendation and absorbance was measured at the same time points as other samples. The results were presented as arbitrary absorbance units.

### Silencing of NOX4 gene

Cells between passages 4 and 10 were transfected with 30 nM siRNA against NOX4 (siNOX4) or non-targeting siRNA (siNeg) (siNOX4#1: 5′GUAUGUUGCAUAACAAGUUtt 3′ (Ambion, Thermo Fisher Scientific); siNOX4#2: 5′GAGAACAGACCUGACUAUGtt 3′ [65]; siNOX4#3: 5′ CAACUCAUAUGGGACAAGAtt 3′; siNeg: negative control #1 (Ambion), using Lipofectamine 2000 (Invitrogen, Thermo Fisher Scientific). 48 h after transfection the medium was replaced by a fresh one and cells were cultured for the subsequent days.

### Quantitative polymerase chain reaction (qPCR)

Total RNA was isolated using the RNeasy Micro Kit (Qiagen), according to the manufacturer's protocol. 500 ng of RNA was used for reverse transcription reaction and its product was mixed with SYBR Green Master Mix (Applied Biosystems, Thermo Fisher Scientific) and 1 μM starters for human NOX4 (forward: 5′ CTCAGCGGAATCAATCAGCTGTG 3′, reverse: 5′ AGAGGAACACGACAATCAGCCTTAG 3′), human ACTB (forward: 5′ CATGTACGTTGCTATCCAGGC 3′, reverse: 5′ CTCCTTAATGTCACGCACGAT 3′), rat Nox4 (forward: 5′GCTTGTTGAAGTATCAAACCAAT 3′, reverse: 5′ TCCAGAAATCCAAATCCAGGT 3′) or rat ACTB (forward: 5′ GGCCAACCGTGAAAAGATGA 3′, reverse: 5′ GACCAGAGGCATACAGGGACAA 3′). The reactions were performed with the use of 7500 Real Time PCR System (Applied Biosystems) and results were analyzed using relative quantification - the ΔΔCt approximation method.

### Detection of senescence-associated β-galactosidase

Detection of senescence-associated β-galactosidase (SA-β-gal) was performed according to Dimri et al. [3] as described previously [31].

### BrdU incorporation assay

To investigate DNA synthesis, 10 μM 5-bromo-2′-deoxyuridine (BrdU; Sigma-Aldrich) was added to culture medium for 18 h. BrdU-positive cells were detected using a primary antibody against BrdU (Becton-Dickinson) and a secondary anti-mouse antibody conjugated with Alexa 488 (Invitrogen). To visualize all nuclei, cells were stained with DAPI (1 μg/ml; Sigma-Aldrich). Slides were examined under the fluorescent microscope (Nikon), photographed using a DS-Fi2 camera (Nikon) and counted using ImageJ software [66].

### Cell cycle analysis

Cells were collected and fixed in 70% ethanol for at least 24 hours in -20°C, incubated in a solution containing 0.2 M sodium hydrogen phosphate and 0.1 M citric acid at pH 7.8, washed in PBS and stained with propidium iodide solution (50 μg/ml) containing sodium citrate (38 mM) and RNase A (500 μg/ml). DNA content was measured by a flow cytometer (FACS Calibur, Becton Dickinson) and analysed using the CellQuest Pro and Modfit software. Each time 10 000 events were collected and analysed.

### ROS content analysis

Cells were collected, counted and their even number (approx. 100 000) was incubated in a serum-free medium contacting 10 μM carboxy-H_2_DCFDA (6-carboxy-2′,7′-dichlorodihydrofluorescein diacetate; Invitrogen), a general oxidative stress indicator, for 30 minutes in 37°C, 5% CO_2_,. Next, cells were washed in PBS, suspended in a probe-free medium and analysed using flow cytometer, FL-2 channel (Becton Dickinson). 30 000 events were collected and analysed using CellQuest Pro software.

### Western blot analysis

The whole cell protein extracts were prepared in the Laemmli buffer [67]. Equal amount of the protein was electrophoretically separated in 8-15% polyacrylamide gels and transferred to nitrocellulose membranes. After blocking in 5% non-fat milk or 5% BSA (Lab Empire) in TBST, membranes were incubated overnight at 4°C with primary antibodies specific for NOX4, ATM, p-ATM Ser1981, p57 (Abcam), p53, p16 (Santa Cruz Biotechnology, Dallas, USA), p-p53 Ser15, ERK1/2, p-ERK1/2 Thr202/Tyr204, HIF1α (Cell Signalling), p21 (Sigma), p27 (BD), GAPDH (Merck Milipore), Rb or cyclin D1 (Neo Markers), washed in TBST and incubated for 1 h in RT with secondary anti-mouse or anti-rabbit antibodies (Dako). For detection, membranes were incubated with ECL, according to the manufacturer's protocol (SuperSignal West Pico Chemiluminescent Substrate, Thermo Fisher Scientific). GAPDH served as a loading control.

### Immunocytochemical detection of 53BP1

Cells were fixed with ice-cold 70% ethanol and stored in -20°C for at least 24 h. Samples were blocked for 30 minutes with 5% BSA in PBS containing 0.5% Tween, 0.1% Triton X-100 (Sigma-Aldrich), washed and incubated for 2 h with a primary antibody against 53BP1 (Novus Biologicals). After washing in PBS, cells were incubated for 1 h with a secondary anti-rabbit antibody conjugated with Alexa 488 and for 15 minutes with DAPI (1 μg/ml) to stain all nuclei. Cells were observed under a fluorescence microscope (Nikon) and photographed. 53BP1 foci were counted and cells were divided into 4 groups: cells without 53BP1 foci in nucleus and cells with 1, 2-5, or more than 5 53BP1 foci per nucleus.

### Immunocytochemical detection of Ki-67 and F-actin

Cells were fixed with 4% paraformaldehyde for 15 minutes, washed in PBS and blocked with 2% BSA and 1.5% goat serum in PBS containing 0.1% Triton X-100. After washing, cells were incubated for 2 h with a primary antibody against Ki-67 (abcam) and then incubated for 1 h with a secondary anti-rabbit antibody conjugated with Alexa 555 and with phalloidin conjugated with FITC to stain F-actin. After washing, all nuclei were stained with DAPI (1 μg/ml). Cells were observed under a fluorescent microscope (Nikon).

### Analysis of senescence-associated secretory phenotype (ELISA assay)

Medium from cell cultures were collected and analysed for the presence of secreted proinflammatory factors, namely interleukin 6 (IL-6), interleukin 8 (IL-8) and vascular endothelial growth factor (VEGF), according to a manufacturer's protocol (R&D Systems). The level of secreted factors was determined with the use of standard curve and normalized to the number of the cells.

### Statistical analysis

The results represent the mean ±SD from at least three independent experiments. Mann-Whitney U test was used to calculate statistical significance: * for *p* < 0.05; ** for *p* < 0.01, *** for *p* < 0.001.

## SUPPLEMENTARY MATERIAL FIGURES


