# Supplementary material for: NOX4 downregulation leads to senescence of human vascular smooth muscle cells
Source: Oncotarget. 2016 Sep 16;7(41):66429–43. doi: 10.18632/oncotarget.12079 (PMC5341811; doi:10.18632/oncotarget.12079)
Supplement: Supplementary file 1 [file oncotarget-07-66429-s001.pdf]

NOX4 downregulation leads to senescence of human vascular smooth muscle cells

Supplementary Material

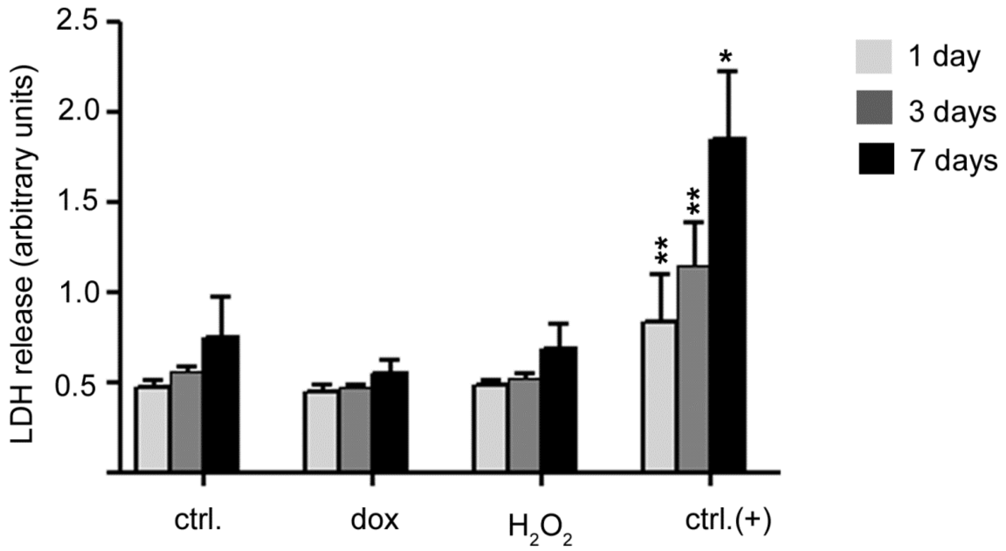

Supplementary Fig.1. VSMCs induced to senescence by doxorubicin or hydrogen peroxide treatment do not lose viability.

Cells were induced to senescence as described in Materials and Methods. For positive control low passage number VSMCs (control cells) were treated with lysis solution according to a manufacturer's recommendation. Graph presents changes in the level of LDH release in the control cells and the cells induced to senescence by dox or H_2O_2 treatment, mean±SD from three independent experiments. For statistical analysis the level of LDH release from dox- or H_2O_2- or lysis solution–treated cells were compared with control cells at each time point.

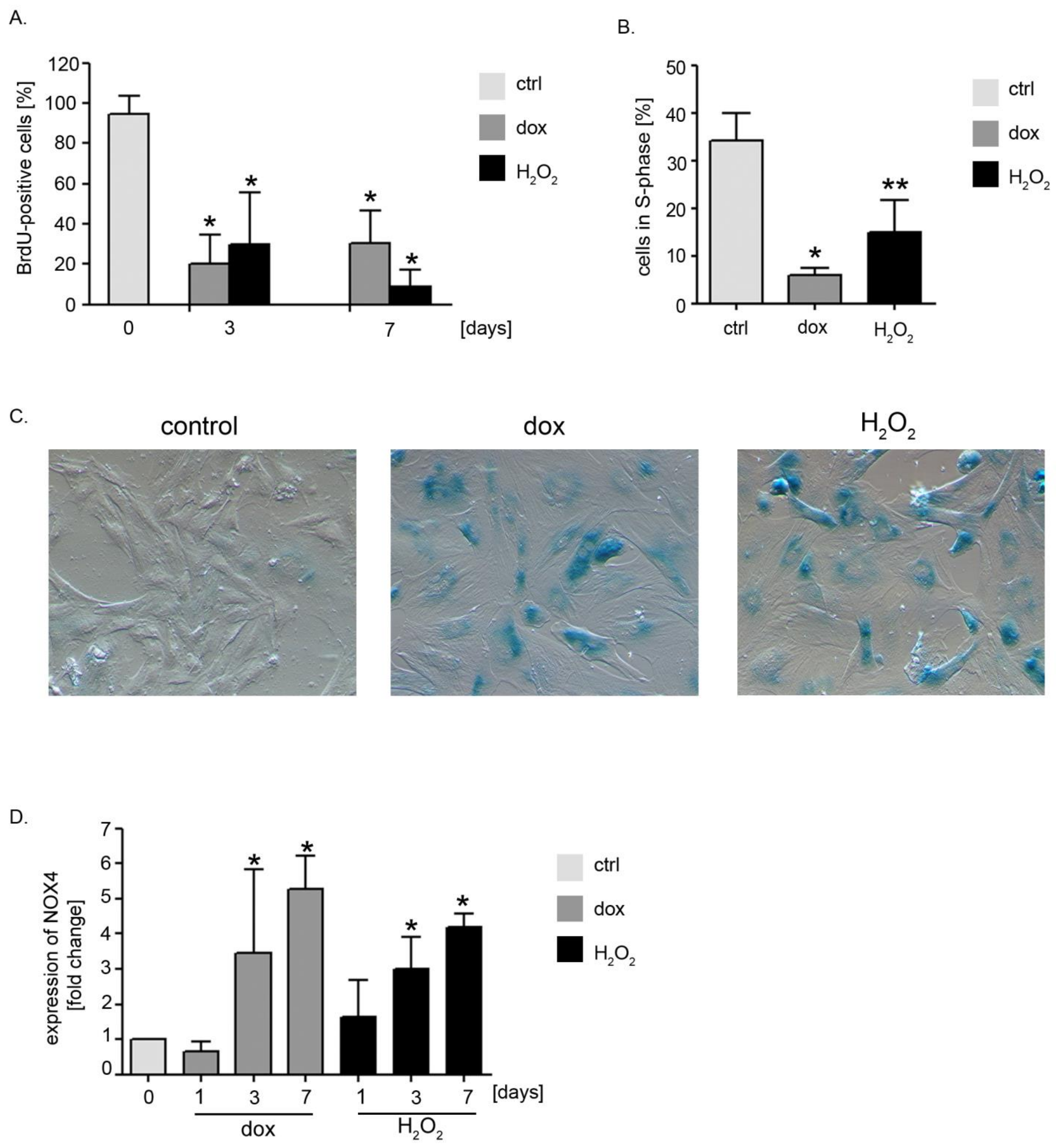

Supplementary. Fig.2 Doxorubicin- or H_2O_2-induced senescence in rat VSMCs.

(A) Analysis of proliferation potential of dox- or H_2O_2-treated cells based on DNA synthesis capacity. Cells were either untreated (ctrl) or treated with dox (1μM for 2 hours) or H_2O_2 (100 μM) and after 6 days BrdU incorporation test was performed; graph presents mean values±SD

from at least three independent experiments. (B) Analysis of the cells in the S-phase of the cell cycle 3 days after treatment with dox or H_2O_2. The mean percentage of cells ±SD in S phase was calculated from three independent experiments using Modfit software. (C) Detection of SA-β-gal activity in non-treated (ctrl), dox- and H_2O_2-treated cells 6 days after treatment. Representative photographs from three independent experiments are shown. (D) The relative level of *Nox4* in the cells undergoing senescence upon dox or H_2O_2 treatment. The level of expression was normalized to *Actb* expression and related to control (non-treated) cells. Graph presents mean expression (±SD) of *Nox4* on the 1st ,3rd and 7th day after transfection measured in at least three independent experiments.

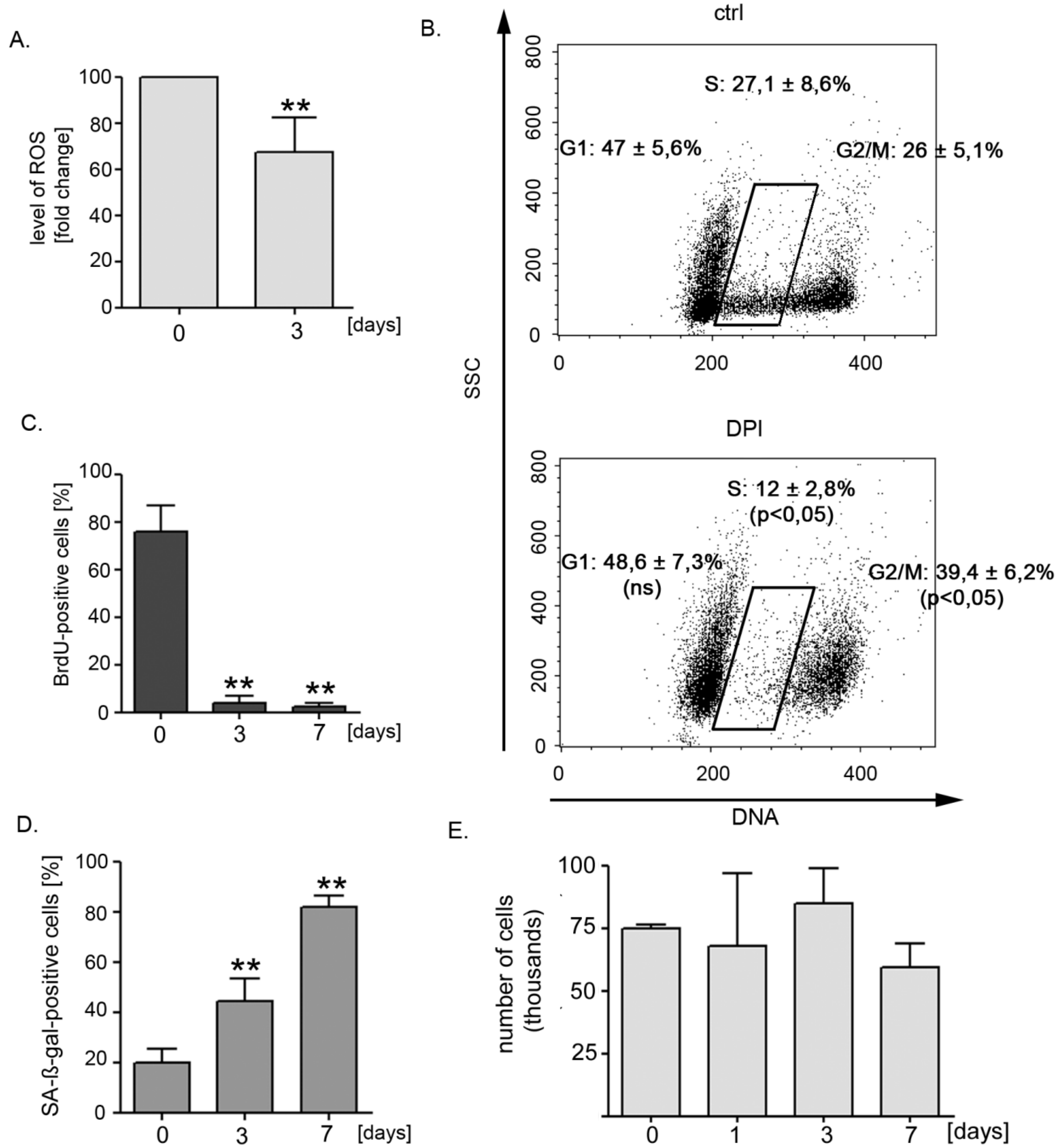

Supplementary Fig.3. Inhibition of NOX by DPI induces senescence of hVSMCs.

(A) The relative level of ROS in hVSMCs after treatment with DPI. Low passage cells (passage number 4-10) were treated with DPI (1.25 μM) for 3 days and the level of ROS was estimated by flow cytometry measurement of carboxy-H_2DCFDA fluorescence. Graph presents the mean

fluorescence intensity (±SD) from six independent experiments of DPI-treated cells in relation to the level of the fluorescence of untreated control cells. (B) Cell cycle analysis of DPI-treated cells, representative dot plots are shown. The mean percentage of cells ±SD in G1, S and G2/M phases calculated using Modfit software from three independent experiments is shown on each plot. (C) Analysis of a proliferation potential estimated on the base of BrdU incorporation test 3 and 7 days after treatment with DPI. Cells were treated with DPI (1.25 μM) for 3 days and then cultured in DPI-free medium. Graph presents mean percentage (±SD) of BrdU-positive cells calculated from five independent experiments. (D) Detection of SA-β-gal activity in control (day 0) and DPI-treated cells for 7 days. The graph presents a mean percentage ±SD of SA-β-gal-positive cells estimated based on six independent experiments. (E) Graph presents changes in the number of cells counted at the 1st, 3rd and 7th day of experiment, mean±SD from 3 independent experiments. The differences in the number of untreated (day 0) and DPI-treated cells (day 1, 3, 7) was statistically insignificant.
